# Hookworm infection causing severe anemia in a stroke patient

**DOI:** 10.11604/pamj.2025.50.60.46842

**Published:** 2025-02-26

**Authors:** Zelalem Mulu Lashitie, Metages Girma Kassahun

**Affiliations:** 1 Department of Internal Medicine, Debre Markos University, Debre Markos, Ethiopia,; 2 Department of Internal Medicine, Felege Hiwot Hospital, Bahirdar, Ethiopia

**Keywords:** Stroke, anemia, hookworm

## Image in medicine

An 82-year-old man has been taking atorvastatin and aspirin after he was diagnosed with ischemic stroke a year ago. He has been experiencing an epigastric burning type of pain, lightheadedness, and significant fatigue for the last three months. He had never experienced hematemesis or melena. Physical examination revealed a BP=120/70 mmHg, PR=108 beats/min, RR=20 breaths/min, and axillary temperature of 37°C. He exhibited pale conjunctiva and non-icteric sclera. The precordial examination was remarkable for ejection systolic murmur at the apex. The abdominal examination and perirectal examination were unremarkable. Neurologic examination revealed hemiparesis of the right extremities (upper motor neuron lesion type of weakness). Laboratory evaluation showed anemia with a hemoglobin of 4.7 gm/Dl and MCV of 57.6 Fl. Peripheral blood smear revealed microcytic, hypochromic RBCs. The stool examination was done 3 times and the 3^rd^ test finally showed ova of hookworms. The stool *Helicobacter pylori* (*H. pylori*) antigen test was negative. The serum creatinine, liver enzymes, and bilirubin levels were all in the normal range. An abdominal ultrasound was normal. The electrocardiogram (ECG) showed sinus tachycardia. Echocardiography was remarkable for grade 2 left ventricular diastolic dysfunction. Later upper gastrointestinal endoscopy showed linear erythema at the antropyloric region, and a motile spiral worm was observed (A and B). He was admitted for a blood transfusion, after which he got 2 units of packed Red blood cells (RBCs). Albendazole was given for 3 days (400 mg orally daily). Proton pump inhibitor (omeprazole 20 mg orally two times daily) was added for the dyspeptic symptoms. Later ferrous sulphate 325 mg tablets were given 3 times daily and aspirin and atorvastatin were continued. He was discharged after a week's stay at the hospital with a pre-discharge hemoglobin of 7.6 gm/Dl. After 2 months, during follow-up, his hemoglobin was 11.3 gm/Dl with significant improvement in anemia symptoms.

**Figure 1:**
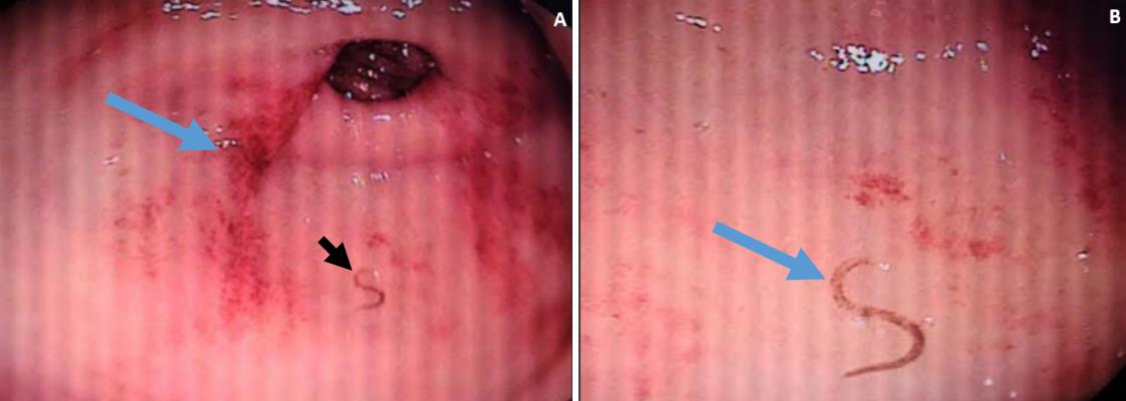
A) linear erythema (blue arrow) at the antropyloric region with a hookworm (black arrow) noted; B) a closer view of hookworm at the antrum (arrow)

